# Uterine Tumor—Death—Autopsy

**Published:** 1860-07

**Authors:** R. F. Adams

**Affiliations:** Amboy, Ill.


ARTICLE 6.

### UTERINE TUMOR—DEATH—AUTOPSY.

BY R. F. ADAMS, M. D., AMBOY, ILL.

April 3, 1860.—Called to see Mrs. P., aged thirty-four years, who was, and had been for the last twelve hours previous, suffering under premonitory symptoms of labor, at her full period of utero-gestation. She informed me that she had a large tumor in her abdomen, occupying the whole of the right side. Parturient pains occurring quite often, I proposed an examination per vaginum, and also over the region of the abdomen, to ascertain the position of the tumor and the gravoid uteris, and the relation, as near as possible, of each to the other.

I found the tumor occupying a portion of the right iliac, the whole of the right lumbar, a portion of the umbilical, and a portion of the right hypochondriac region. The uterus occupied its original position, seemingly, with the exception of being thrown somewhat to the left side. Through the parieties of the abdomen I followed the margin of the tumor, to its connection with the uterus. I endeavored to insinuate my fingers between the tumor and uterus by pressing upon the abdominal parieties, but I could scarcely feel the existence of a fissure between the two. In fact, from the most careful examinations I could make, it seemed to me that the tumor was continuous with the uterus, by a very broad and extensive connection with the fundus of that organ.

Some twelve hours having elapsed since my first examination per vaginum, in which I discovered the os tincæ beginning to dilate very slightly. Pains for the last two hours having increased in frequency and severity, I made another examination, and found the os uteri dilated some two inches in diameter, the membranes protruding, and labor progressing as in ordinary cases. I now sought for the presentation of the fœtus. I passed my fore-finger around the membranes and into the os uteri, where it met the left foot of the fœtus. Traversing still farther with my finger, I soon discovered the other foot, and after some two hours more, in which I watched the case with the most intense anxiety for the safety of both mother and child, I was enabled to traverse the anterior portion of the breech of the fœtus, its genital organs, with its knees coming down upon the arch of the pubes, and its feet presenting at the vulva. With the membranes yet entire, and the bag of waters quite protruding from the vulva, I ruptured them, and sought to return the feet and bring down the breech into the proper axis. Finding I could not do this, I brought down the feet, disengaged the knees from the arch of the pubes, and delivered my patient of a fine boy nearly in an asphyxiated condition; but by the most assiduous efforts, I succeeded in restoring it to active life. On the expulsion of the head, a free rush of blood followed for a moment. In some twenty minutes the placenta came down all right, and my patient was very easy and comfortable. My attention was now anxiously drawn to the uterus. I found the tumor occupying a new position, having dropped nearly into the space just previously occupied by the uterus. I made search for the uterus below the tumor, as a distinct organ, but could only find it from the most careful manipulations I could make, continuous with the tumor, though contracted so firmly as to show that nature's work was so far completed favorably. The tumor now occupied the right iliac, the hypogastric, a portion of the left iliac, and lower half of the umbilical regions. Fearing too much pressure upon the uterus from the weight of the tumor, I applied a bandage over the abdomen in a manner to sustain a portion of its weight, gave my patient an anodyne, and left her in a very favorable condition. For two days she continued very comfortable and cheerful, and everything appeared to indicate a fortunate tendency. On the third day, however, my fears began to approach a dread reality that my patient must die. She began to complain of pains in the region of the left ovary, which soon spread over the hypogastric region, and to the right ovary. Every means in my power was put in force to arrest the progress of inflammatory action and restore her to a comfortable condition, but to no purpose. Death closed her sufferings on the morning of the sixth day after her confinement.

*Autopsy twelve hours after Death.*—The appearance of the abdominal parieties indicated a subject at full time of utero-gestation.

I commenced my first incision in the linea alba, at a point three inches above the umbilicus, and continued it down nearly to the symphysis pubis. I then made my second and last, from the umbilicus outward to the right side, which gave me a full view of the tumor and organs *in situ.* The tumor was now to be seen occupying the hypogastric, the lower two-thirds of the umbilical, and a good portion of the right iliac region. The small intestines and the ascending portion of the colon were pushed far to the left and upward from their natural position. On raising the tumor from its position, I found its growth continuous with the entire fundus of the uterus and the broad ligament and ovary of the right side. The parieties of the fundus of the uterus were very much increased in thickness, as also were the broad ligaments and ovaries of both sides. All these organs were found in a sphacelated condition. In dissecting the tumor from the fundus of the uterus, the parieties of the latter were found to be in attachment with the former, a thickness of one and three-fourths inches from the margin of its cavity outward, anteriorly and posteriorly and laterally to the left side, and continuous to the right, embracing the broad ligament and ovary. On removing the tumor its weight was found to be ten and a half pounds. It was mostly of an organization resembling indurated liver, with portions somewhat softer, indicating a gelatinous or cheese-like substance. The blood-vessels of this morbid growth were enormous in size and immense in number, and all derived from the uterus.

It is proper to remark, in connection with this article, that the deceased had been apprised, for years, by the best medical authority in New England, that death would sooner or later supervene from this morbid growth. She had expressed to her relatives her desire, that when that time should come, an autopsy should be had, and the facts given to the medical profession for the benefit of humanity.

The tumor will be deposited in the museum of Rush Medical College, where all interested in pathological and surgical investigations can examine it.

